# The Present Status of Medical Science Concerning the Causes and Prevention of Epidemic Diseases

**Published:** 1877-05

**Authors:** N. S. Davis


### THE PRESENT STATUS OF MEDICAL SCIENCE CONCERNING THE CAUSES AND PREVENTION OF EPIDEMIC DISEASES.

By N. S. DAVIS, M. D.

[Read to the Evanston Philosophical Association, Feb. 12th, 1877.

In a former paper read to this society, I endeavored to explain the true nature and objects of medical science. It was then claimed that medical science was not a compact aggregation or embodiment of philosophical theories, or dogmas, concerning the nature of disease, and the modus operandi of medicines; but rather an appropriation of the facts and principles of all the physical and natural sciences, so far as they can be made to reveal the causes, nature and tendencies of diseases, and the agents capable of acting either as preventives or curatives. The physician studies the human system in the same manner, and by means of the same instruments and appliances, that the thorough naturalist studies any other species of animal. He analyzes, or dissects, by means of the scalpel and microscope, all the structures of the body, to ascertain the existence and extent of each structure and organ, and the relations of each part to all the rest. By simple observation and experiment, he endeavors to ascertain the exact function or office performed by each part, and the influence it has over the action of other parts. Having, in this manner, become familiar with the anatomy and physiology of the human system, as a living organization, he endeavors, by the same methods of inquiry, to ascertain in what way the various structures and functions may be altered from their natural conditions, so as to constitute disease. For the latter, instead of being some mysterious entity, or substance, or potency that enters the human system as an enemy, and is to be met, driven out, or neutralized by some specific remedy, administered in accordance with some supposed universal law of cure, is simply a deviation from the natural or healthy standard of action in some structure or organ. Such deviation may consist of increased, diminished or perverted actions, according to the nature of the exciting cause or causes.

And the application of remedies must be governed by the same principles of reason, or common sense, as govern us in all other things. If the morbid action is one of increase or excitement, such remedies should be chosen as would repress or diminish it; if of diminution, such as would tend to sustain or increase it; or if perverted, they should be such as would correct it. And in all cases, the causes that have been operative in producing the morbid conditions, should be removed as far as practicable. And this brings us to one of the leading questions for discussion this evening, namely, how far does medical science, or the present state of human knowledge, reveal to us the exact causes of the more important acute, or endemic and epidemic diseases, such as typhoid, typhus and scarlet fevers, diphtheria, yellow fever, plague, cholera, &c. To the non-professional mind, and even to many of the less thoughtful in the profession, it may seem a very simple and easy matter to determine the cause or causes giving rise to any particular form of disease.

And if we could rely on the dogmatic assertions of closet speculators and partial observers, we might have every question of causation settled, at least, until it was contradicted by the next theorist.

•One of the most transcendental speculators of a past generation, confidently asserted that all chronic diseases, at least, were derived from syphilis, sycosis and itch.

Unfortunately, however, he left us entirely in the dark as to what caused either of this prolific trio of ailments. Another more ignorant founder of a special sect, based his whole system of medicine on two very simple dogmas. The first was that *heat* is life and *cold* is death. The second, that minerals exist in the earth, gravitate downwards, and, if used, would pull men after them into the earth; while vegetables grow upward, symbolical of life, and are, therefore, the only proper remedies. So simple a basis of course required nothing more than steam, Cayenne pepper and "number six" for its superstructure of medication. But if this was the absurd deduction of an ignoramus, not very unlike it have been the theoretic deductions of many who have occupied high places in the profession, and whose writings constitute an important part of our medical literature.

Take, for instance, the theory of Broussais and Rush, that all disease is primarily irritative or excitant, consequently originating from causes of an irritant or stimulating character, and requiring sedative or depressing agents as remedies. Or that of Dr. Chambers, presenting just the opposite view, namely, that all disease is a diminution of life; and, therefore, that all remedial agencies must be such as are calculated to excite or sustain life. Or, to come still closer to the present time, the recent general application of the microscope, in the study of all departments of physical science, and among them those of anatomy, physiology, pathology and etiology, has brought out to view, substantially, a new kingdom of nature, consisting of vegetable and animal germs and organizations pervading the air, the water, and almost everything else that is capable of being brought under the field of the microscope.

As might have been expected from the well-known tendency of the human mind to make coincidences stand in the relation of causes and effects, and to deduce conclusions from partial or incomplete observation of facts, the discovery of some variety of organic germs in the evacuations of cholera patients, in the air and water of malarious districts; in the blood of those laboring under syphilis, scarlet fever, &c.; and in the membranous exudations of diptheria, thrush, and various cutaneous diseases, led to the confident assertion of each successive observer, that the co-existence of these germs was sufficient proof that they constituted the efficient cause of whatever disease they were found associated with. Hence the rapid development and popularization of the germ theories of causation of diseases, and especially of all those of an acute endemic or epidemic character.

As similar germs to those found in connection with certain diseases are known to be developed and multiplied by the chemical process of fermentation, so their multiplication in the human system, or its evacuations, is supposed to be carried on by similar processes. Hence the grouping of a large number of diseases under the general terms, zymotic, or infectious. Assuming the zymotic and germ theories in regard to the efficient causes of disease, it was a natural and not illogical inference that the diseases so caused would be propagated by the reproduction and multiplication of the infectious germs in some one or all of the emanations or evacuations from the bodies of the sick. Hence, to destroy these emanations, or so modify them as to prevent the fermentation and evolution of germs, became one of the chief means for preventing the propagation and spread of diseases. Thus, no sooner had Doctor A. promulgated his discovery of certain supposed peculiar microscopic germs in the evacuations of patients affected with epidemic cholera, than these evacuations were made the medium of spreading the disease throughout the world, by being thrown into privies, mingled with surface water, attached to soiled clothing, and deposited in unsuspected places by travelers. And the well-known class of antiseptics and disinfectants were speedily brought into requisition to intercept the fermentive processes, and thereby render the dreaded evacuations harmless. The same results followed the adoption of the germ theory in relation to all other diseases. Hence, in the many recent letters and discussions in the Chicago daily press, concerning the prevalence of scarlet fever and diphtheria, three out of five of the writers based their thoughts and remedies on the assumption that these diseases were caused by zymotic or germ propagation, and proposed some kind of antiseptic, like the carbolates, sulpho-carbolates, permanganates, chlorates, or sulphites, either as remedies or preventatives, or both.

But what evidence have we that any form of epidemic disease is, or has been, caused by organic germs, either vegetable or animal? I answer, just the same evidence that Samuel Thompson had, that heat is life and cold is death, namely, simple coincidence, nothing more, nothing less. That certain organic germs were discovered in epidemic cholera evacuations, especially after they had been allowed to stand several hours, is true. I have seen the same myself; but that does not prove that they produced the disease.

On the contrary, more extended observation developed the fact that germs of the same identical character were discoverable in the evacuations of ordinary cholera morbus, summer complaints of children, and probably in any serous evacuation from mucous membranes, if treated in the same manner. There is no doubt but Doctor B. discovered organic germs in the blood of his patient laboring under syphilis, and he would have probably discovered the same bodies in the blood of any of his patients not affected with syphilis, had he taken the trouble to have extended his observations that far, before hastening to his conclusion that they were the cause of that vile disease.

The same remarks may be made in regard to the propagation of scarlet fever, diphtheria, &c., by fermentation or germ evolution. Indeed, the whole theory of zymosis and germ developments, as causes of disease, must be regarded in the present state of our knowledge, as only another specimen of hasty generalization, or the deduction of important conclusions from a very incomplete observation of facts.

And the same remark would be true, if applied to all the claims hitherto made in regard to the efficacy of particular remedies as preventives of disease. Take, for instance, the sulphites, sulpho-carbolates, or belladonna, as remedies administered for the prevention of scarlet fever. Most of you might suppose it a very easy matter to determine their value by simple trial in a given number of cases. And so it would be, if it were a fact that every child unprotected, when exposed to scarlet fever, actually took the disease. But such is not the fact. On the contrary, quite a large percentage of those exposed in every epidemic escape an attack. During the present season, I have been personally acquainted with two large families, each closely connected with quite a circle of other families, in each of which a single child was attacked with severe scarlet fever. The well children were simply kept from free intercourse with the sick ones, the house and sick rooms kept well ventilated and cleanly, and not another child in either family took the disease. Now, if, when the disease first appeared in these families, suitable doses of belladonna or sulpho-carbolates had been given to each of the well children, while the disease remained in the respective houses, and no one had taken it, doubtless both the families, their friends, and perhaps the attending physician, would have deemed their exemption as full proof of the efficacy of the preventive remedy administered, And yet, you perceive that such a conclusion would have been entirely fallacious. It is on just this kind of evidence, however, that the reputation of all the so-called preventive medicines rests. To determine the protective value of any particular remedy, it is necessary that careful observations and records be made during the prevalence of several epidemics of scarlet fever, to ascertain first the ratio of those who escape attacks after exposure, without the use of any drug; and then a parallel series of observations and records in relation to the ratio of those who escape attacks after exposure, under the use of some particular remedy.

It will be obvious to you, that such a mode of investigation would require both time, and more opportunities for observation than often fall to the lot of a single practitioner; and hence to make it entirely reliable, would require the co-operation of several practitioners occupying different localities; but subjecting the results of their observations to a careful comparison with each other.

It must be acknowledged that but few attempts have been made to determine the value of preventive medicines in the manner here indicated. But so far as such attempts have been carried, they have not sustained the reputation of any remedy as a reliable prophylactic.

To those who have given little thought to the subject, it may appear surprising that so little is known definitely in regard to the direct exciting or essential causes of diseases. But no such surprise will be manifested by those who comprehend the complexity of the problems involved.

The human system itself is an exceedingly complex organization, made up of a variety of delicate textures, and a series of organs, the functions of each bearing an important relation to all the rest, and the whole undergoing constant molecular change in two opposite directions, the one carrying the food and drink we take through a succession of changes, until it is converted into blood and flesh, the other converting the materials of the tissues into effete or waste matter, returning it into the blood, to be carried to the organs designed for its elimination, such as the skin, kidneys and lungs. In addition, every part of this complex machinery is pervaded by a system of nerves, delicately sensitive to every impression, either from within or without. If we reflect that this complicated mechanism called man, is continually in contact with, and pervaded by, all the imponderable forces, such as heat, electricity, &c., and surrounded by an atmosphere not only composed of its fixed elements of oxygen and nitrogen, but containing in addition an ever varying quantity of light, heat, electricity, aqueous vapor, and an almost endless variety of emanations, both organic and inorganic, from the surface of the earth, we shall see that every question relating to the action of particular agents as causes of disease, is sufficiently complex to require the greatest care to avoid the adoption of deceptive and erroneous conclusions.

Another difficulty in studying the causes of epidemic diseases arises from the fact that all of them are irregular in the time of their appearance, and temporary in their duration. Hence, unless a permanent system of observation and record is established in a large number of localities, embracing a daily record of the atmospheric conditions relating to heat, pressure, moisture, electricity, ozone, and direction and velocity of winds; microscopic examinations of the air and water; and a careful record of the date of attack of all diseases, the causes of which are in question, we cannot have in any given epidemic the data necessary for determining what germs, substances, or conditions are peculiar to that epidemic, and what are simply coincident. It will be evident to you all that the continuous co-operation of the meteorologists, microscopists and practising physicians, in such parallel courses of observation, through a series of years, is a work of no little magnitude and difficulty. And yet so long as we are without the results of such observation, we are certainly without the data necessary for determining the essential causes of epidemic, and even endemic, diseases. Again, the well-known temporary duration of epidemic diseases in any given locality or community often leads to errors in regard to the efficiency of sanitary regulations. Almost every epidemic has its period of rise, climax and decline.

Hence if it so happens that after an epidemic of scarlet fever, for instance, has progressed until public attention is aroused and the epidemic has reached its climax, as is usually the case, some special sanitary measures are adopted, the disease soon begins to decline, and in a few weeks disappears. Of course the sanitary measures adopted receive the credit of having "stamped out the disease," when, in truth, it had disap peared in strict accordance with its own law of decline. Perhaps you are ready to ask, then, if so little is known concerning the essential causes of disease, and medical literature is so full of errors and false conclusions—of what value is medical science to the community; or rather, why call it a science? I answer that all these theoretic dogmas and errors arising from partial observation of facts, bear the same relation to medical science that the plutonic, neptuvian, and glacial theories do to geology—or the speculations of a Darwin and a Huxley do to anthropology and natural history. While the advocates of these theories are amusing the world with their grotesque mixtures of facts and fancies, the great body of geologists and students of natural history are steadily adding fact to fact, enlarging the boundaries, and extending the usefulness of their respective departments of science. So the great body of medical observers have been, and still are, steadily accumulating the facts and perfecting the art of medicine. And while the collateral sciences of physical geography, meteorology, organic chemistry, and microscopy have not yet enabled them to identify the essential causes of epidemics, they have, nevertheless, acquired a mass of facts relating to the circumstances and influences, that favor the development, propagation and malignancy of such diseases, of the highest value to mankind. The results obtained may be briefly expressed in the following propositions:

1st. That confined and impure air; water impregnated with organic matter; unwholesome and insufficient food; uncleanliness of person and premises; and depressing mental influences directly favor the development, propagation and severity of all epidemic and infectious diseases.

2d. That so far as these conditions can be prevented, or removed when they exist, in the same proportion will we limit the amount, and lessen the severity of the diseases to which we have alluded. It is on these propositions that all those modern sanitary measures, embraced under the heads of ventilation, cleanliness, sewerage, water supply, and wholesome food, which have so markedly diminished the ratio of sickness and mortality in many of the most populous communities in Christendom, are founded. Many generations may yet pass before medical men can identify and study the exact poison or agent, whether organic or inorganic, that constitutes the efficient cause of scarlet fever, or any other epidemic disease; and we may expect little benefit from specific medicines given for the purpose of neutralizing or destroying such supposed cause. But every carefully observed fact that enables us to see more clearly the conditions which favor the development, spread, and severity of any given disease, will be of value in suggesting, to the thoughtful and well-disciplined mind, practical measures for modifying or preventing those conditions.

Such, we regard as the present status of medical science in relation to the essential causes of epidemic diseases,and the attempts at prevention by specific medication.

